# Crystal structure of alluaudite-type NaMg_3_(HPO_4_)_2_(PO_4_)

**DOI:** 10.1107/S205698901501155X

**Published:** 2015-06-20

**Authors:** Ahmed Ould Saleck, Abderrazzak Assani, Mohamed Saadi, Cyrille Mercier, Claudine Follet, Lahcen El Ammari

**Affiliations:** a Laboratoire de Chimie du Solide Appliquée, Faculté des Sciences, Université Mohammed V, Avenue Ibn Battouta, BP 1014, Rabat, Morocco; b Laboratoire des Matériaux Céramiques et Procédés Associés, EA2443, Université de Valenciennes et du Hainaut-Cambrésis, Boulevard Charles de Gaulle, BP 59600, Maubeuge, France

**Keywords:** crystal structure, transition metal phosphates, alluaudite structure type, hydro­thermal synthesis, hydrogen bonding

## Abstract

The title compound, sodium trimagnesium bis­(hydrogen phosphate) phosphate, was obtained under hydro­thermal conditions. In the crystal, two types of [MgO_6_] octa­hedra, one with point group symmetry 2, share edges to build chains extending parallel to [10-1]. These chains are linked together by two kinds of phosphate tetra­hedra, HPO_4_ and PO_4_, the latter with point group symmetry 2. The three-dimensional framework delimits two different types of channels extending along [001]. One channel hosts the Na^+^ cations (site symmetry 2) surrounded by eight O atoms, with Na—O bond lengths varying between 2.2974 (13) and 2.922 (2) Å. The OH group of the HPO_4_ tetra­hedron points into the other type of channel and exhibits a strong hydrogen bond to an O atom of the PO_4_ tetra­hedron on the opposite side.

## Abstract 2

NaMg_3_(PO_4_)(HPO_4_)_2_ crystallizes in the alluaudite-type structure. Two types of [MgO_6_] octa­hedra, one [NaO_10_] polyhedron, one orthophosphate and one hydrogenphosphate tetra­hedron form the structural set-up.

## Chemical context   

By means of hydro­thermal processes (Demazeau, 2008 ▸; Yoshimura & Byrappa, 2008 ▸), we have previously succeeded in the isolation of the mixed-valence manganese phosphates *M*Mn^II^_2_Mn^III^(PO_4_)_3_ (*M* = Ba, Pb, Sr) adopting the α-CrPO_4_ structure type (Assani *et al.*, 2013 ▸; Alhakmi *et al.*, 2013*a* ▸,*b* ▸). In addition, within the pseudo-ternary systems Ag_2_O–*M*O–P_2_O_5_, hydro­thermal syntheses have allowed us to obtain other α-CrPO_4_ isotype phosphates, *viz*. Ag_2_ *M*_3_(HPO_4_)(PO_4_)_2_ (*M* = Co, Ni) while AgMg_3_(HPO_4_)_2_(PO_4_) is found to adopt the alluaudite structure type (Assani *et al.*, 2011*a* ▸,*b* ▸,*c* ▸). Other hydro­thermally grown phosphates with the alluaudite structure include AgCo_3_(HPO_4_)_2_(PO_4_) (Guesmi & Driss, 2002 ▸), AgNi_3_(HPO_4_)_2_(PO_4_) (Ben Smail & Jouini, 2002 ▸), *A*Mn_3_(HPO_4_)_2_(PO_4_) (*A* = Na, Ag) (Leroux *et al.*, 1995*a* ▸,*b* ▸) and NaCo_3_(HPO_4_)_2_(PO_4_) (Lii & Shih, 1994 ▸). Phosphates belonging to either the α-CrPO_4_ or alluaudite structure type or derivatives thereof are still in the focus of research owing to their promising applications as battery materials (Trad *et al.*, 2010 ▸; Essehli *et al.*, 2015*a* ▸,*b* ▸; Huang *et al.*, 2015 ▸).

The crystal structures of alluaudite-type phosphates exhibit channels in which the monovalent cations are localized. Indeed, this is strongly required for conductivity properties. The crystal structure of alluaudite can be formulated by the general formula (*A*1)(*A*2)(*M*1)(*M*2)_2_(PO_4_)_3_, (Moore & Ito, 1979 ▸). The two *A* sites can be occupied by either mono- or divalent medium-sized cations while the two *M* cationic sites correspond to an octa­hedral environment generally occupied by transition metal cations. On the basis of literature research, it has been shown that the hydro­thermal process allows, in general, stoechiometric phases to be obtained while solid-state reactions give rather a statistical distribution of cations on either the *A* or *M* sites, leading to non-stoechiometric compounds (Bouraima *et al.*, 2015 ▸; Khmiyas *et al.*, 2015 ▸).

In line with our focus of inter­est, we hydro­thermally synthesized the compound NaMg_3_(PO_4_)(HPO_4_)_2_ and report here its crystal structure.

## Structural commentary   

**Figure 1:**
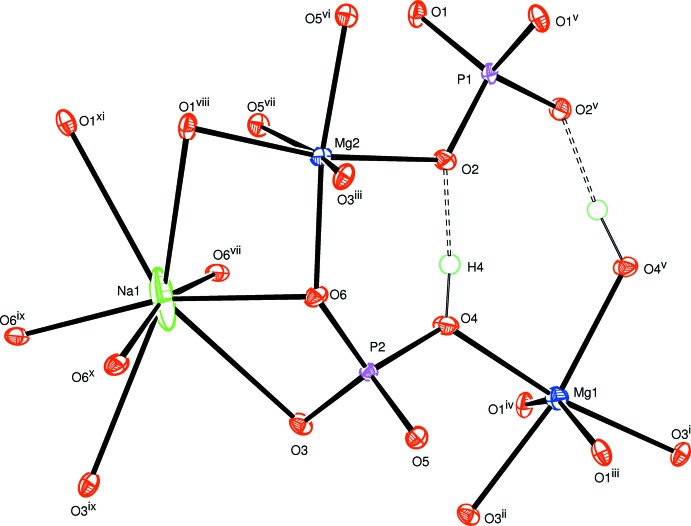
The principal building units in the structure of the title compound. Displacement ellipsoids are drawn at the 50% probability level. Hydrogen bonds are indicated by dashed lines [Symmetry codes: (i) *x* + 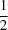, *y* + 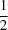, *z*; (ii) −*x* + 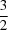, *y* + 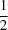, −*z* + 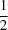; (iii) −*x* + 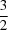, −*y* + 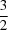, −*z* + 1; (iv) −*x* + 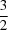, −*y* + 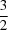, −*z*; (v) −*x* + 1, −*y* + 1, −*z*; (vi) −*x* + 1, *y*, −*z* + 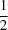; (vii) *x*, −*y* + 1, *z* + 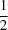; (viii) *x* − 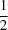, −*y* + 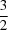, *z* − 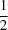; (ix) −*x* + 2, *y*, −*z* + 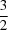; (x) −*x* + 2, −*y* + 1, −*z* + 1; (xi) *x* + 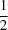, −*y* + 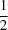, *z* + 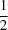; (xii) −*x* + 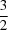, −*y* + 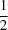, −*z* + 1; (xiii) *x*, −*y* + 1, *z* − 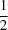.]

**Figure 2:**
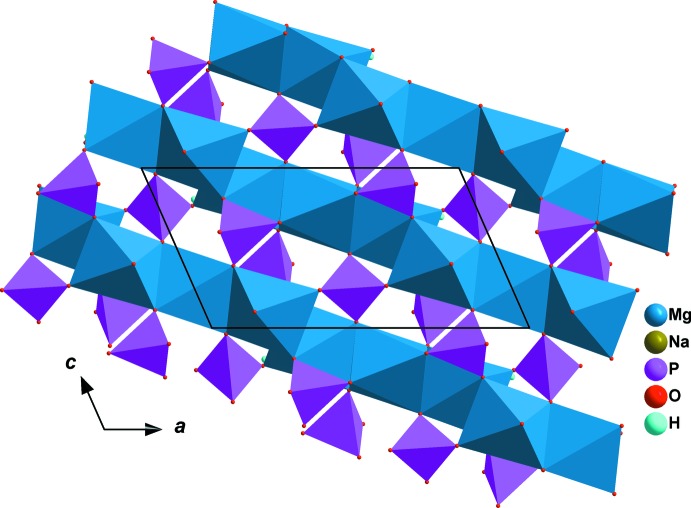
A sheet resulting from the linkage of kinked chains *via* vertices of PO_4_ tetra­hedra.

**Figure 3:**
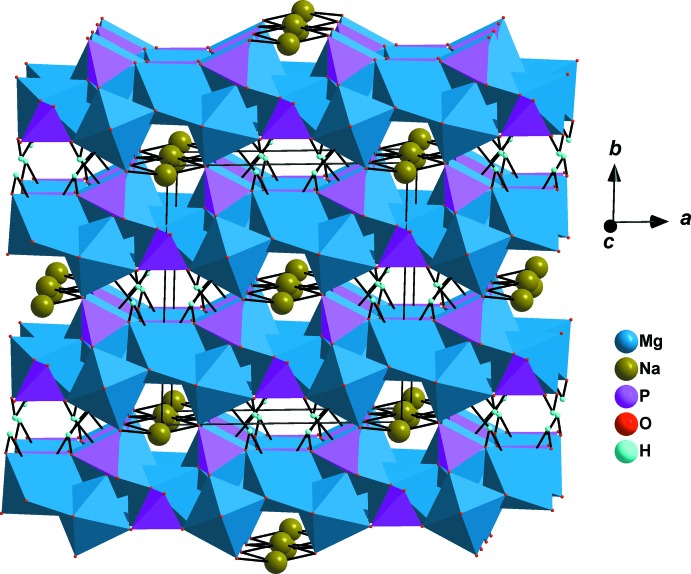
Polyhedral representation of the NaMg_3_(HPO_4_)_2_(PO_4_) structure showing channels along [001]. The O—H⋯O hydrogen bonds are indicated by dashed lines.

The principal building units of the allaudite structure of the title compound are represented in Fig. 1 ▸. The three atoms Mg1, Na1 and P1 are located on a twofold rotation axis (Wyckoff position 4*e*). Selected inter­atomic distances are compiled in Table 1 ▸. The three-dimensional framework of this structure consists of kinked chains of edge-sharing MgO_6_ octa­hedra running parallel to [10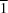]. The chains are held together by regular P1O_4_ phosphate groups, forming sheets perpendicular to [010], as shown in Fig. 2 ▸. The stacked sheets delimit two types of channels along [001]. One of the channels is occupied by Na^+^ cations surrounded by eight oxygen atoms (Table 1 ▸), whereas the second channel contains the hydrogen atoms of the HP2O_4_ tetra­hedra, as shown in Fig. 3 ▸. They form strong hydrogen bonds (Table 2 ▸, Figs. 1 ▸ and 3 ▸) with one of the oxygen atoms of PO_4_ tetra­hedra on opposite sides.

## Synthesis and crystallization   

Colourless parallelepiped-shaped crystals of the title compound were grown under hydro­thermal conditions, starting from a mixture of Na_4_P_2_O_7_·10H_2_O, MgO and H_3_PO_4_ (85 wt%) in the molar ratio Na_4_P_2_O_7_·10H_2_O:MgO:H_3_PO_4_ = 1:3:3. The hydro­thermal reaction was conducted in a 23 ml Teflon-lined autoclave, filled to 50% with distilled water and under autogenous pressure at 483 K for four days.

## Refinement   

Crystal data, data collection and structure refinement details are summarized in Table 3 ▸. The minimum and maximum electron densities are located 0.71 and 0.17 Å from O5 and H4, respectively. The O–bound H atom was initially located in a difference map and refined with an O—H distance restraint of 0.93 Å, and with *U*_iso_(H) = 1.5*U*_eq_(O).

## Supplementary Material

Crystal structure: contains datablock(s) I. DOI: 10.1107/S205698901501155X/wm5174sup1.cif

Structure factors: contains datablock(s) I. DOI: 10.1107/S205698901501155X/wm5174Isup2.hkl

CCDC reference: 1406819

Additional supporting information:  crystallographic information; 3D view; checkCIF report

## Figures and Tables

**Table 1:** Selected bond lengths ()

Mg1O3^i^	2.1224(13)	Na1O3	2.8840(19)
Mg1O1^ii^	2.1312(12)	Na1O1^vii^	2.922(2)
Mg1O4	2.1669(14)	P1O1^viii^	1.5372(12)
Mg2O6	2.0234(13)	P1O1	1.5372(12)
Mg2O3^ii^	2.0686(13)	P1O2^viii^	1.5476(13)
Mg2O2	2.0696(14)	P1O2	1.5476(13)
Mg2O5^iii^	2.0729(13)	P2O5	1.5234(12)
Mg2O5^iv^	2.0955(13)	P2O6	1.5263(12)
Mg2O1^v^	2.1153(14)	P2O3	1.5349(13)
Na1O6	2.2974(13)	P2O4	1.5806(13)
Na1O6^vi^	2.4386(13)		

Symmetry codes: (i) 
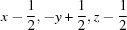
; (ii) 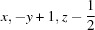; (iii) 
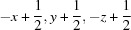
; (iv) 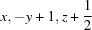; (v) 
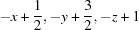
; (vi) 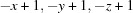; (vii) 
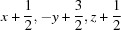
; (viii) 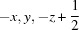.

**Table 2:** Hydrogen-bond geometry (, )

*D*H*A*	*D*H	H*A*	*D* *A*	*D*H*A*
O4H4O2	0.93	1.57	2.4932(17)	174

**Table 3:** Experimental details

Crystal data	
Chemical formula	NaMg_3_(HPO_4_)_2_(PO_4_)
*M*_r_	382.85
Crystal system, space group	Monoclinic, *C*2/*c*
Temperature (K)	296
*a*, *b*, *c* ()	11.8064(6), 12.0625(7), 6.4969(4)
()	113.805(2)
*V* (^3^)	846.54(8)
*Z*	4
Radiation type	Mo *K*
(mm^1^)	1.06
Crystal size (mm)	0.36 0.24 0.18

Data collection	
Diffractometer	Bruker X8 APEX
Absorption correction	Multi-scan (*SADABS*; Bruker, 2009 ▸)
*T*_min_, *T*_max_	0.504, 0.748
No. of measured, independent and observed [*I* > 2(*I*)] reflections	9797, 1291, 1138
*R*_int_	0.038
(sin /)_max_ (^1^)	0.714

Refinement	
*R*[*F* ^2^ > 2(*F* ^2^)], *wR*(*F* ^2^), *S*	0.025, 0.072, 1.09
No. of reflections	1291
No. of parameters	88
H-atom treatment	H-atom parameters constrained
_max_, _min_ (e ^3^)	0.57, 0.54

Computer programs: *APEX2* and *SAINT* (Bruker, 2009 ▸), *SHELXS* (Sheldrick, 2008 ▸), *SHELXL2013* (Sheldrick, 2015 ▸), *ORTEP-3 for Windows* (Farrugia, 2012 ▸). *DIAMOND* (Brandenburg, 2006 ▸) and *publCIF* (Westrip, 2010 ▸).

## supplementary crystallographic information

### Crystal data

**Table:**

NaMg_3_(HPO_4_)_2_(PO_4_)	*F*(000) = 760
*M**_r_* = 382.85	*D*_x_ = 3.004 Mg m^−^^3^
Monoclinic, *C*2/*c*	Mo *K*α radiation, λ = 0.71073 Å
*a* = 11.8064 (6) Å	Cell parameters from 1291 reflections
*b* = 12.0625 (7) Å	θ = 2.5–30.5°
*c* = 6.4969 (4) Å	µ = 1.06 mm^−^^1^
β = 113.805 (2)°	*T* = 296 K
*V* = 846.54 (8) Å^3^	Block, colourless
*Z* = 4	0.36 × 0.24 × 0.18 mm

### Data collection

**Table:**

Bruker X8 APEX diffractometer	1291 independent reflections
Radiation source: fine-focus sealed tube	1138 reflections with *I* > 2σ(*I*)
Graphite monochromator	*R*_int_ = 0.038
φ and ω scans	θ_max_ = 30.5°, θ_min_ = 2.5°
Absorption correction: multi-scan (*SADABS*; Bruker, 2009)	*h* = −16→16
*T*_min_ = 0.504, *T*_max_ = 0.748	*k* = −17→17
9797 measured reflections	*l* = −9→8

### Refinement

**Table:**

Refinement on *F*^2^	0 restraints
Least-squares matrix: full	Hydrogen site location: difference Fourier map
*R*[*F*^2^ > 2σ(*F*^2^)] = 0.025	H-atom parameters constrained
*wR*(*F*^2^) = 0.072	*w* = 1/[σ^2^(*F*_o_^2^) + (0.0362*P*)^2^ + 1.4203*P*] where *P* = (*F*_o_^2^ + 2*F*_c_^2^)/3
*S* = 1.09	(Δ/σ)_max_ < 0.001
1291 reflections	Δρ_max_ = 0.57 e Å^−^^3^
88 parameters	Δρ_min_ = −0.54 e Å^−^^3^

### Special details

**Table:**

Geometry. All e.s.d.'s (except the e.s.d. in the dihedral angle between two l.s. planes) are estimated using the full covariance matrix. The cell e.s.d.'s are taken into account individually in the estimation of e.s.d.'s in distances, angles and torsion angles; correlations between e.s.d.'s in cell parameters are only used when they are defined by crystal symmetry. An approximate (isotropic) treatment of cell e.s.d.'s is used for estimating e.s.d.'s involving l.s. planes.

### Fractional atomic coordinates and isotropic or equivalent isotropic displacement parameters (Å^2^)

**Table:**

	*x*	*y*	*z*	*U*_iso_*/*U*_eq_	
Mg1	0.0000	0.27947 (7)	0.2500	0.00857 (18)	
Mg2	0.29000 (6)	0.66219 (5)	0.37489 (10)	0.00643 (14)	
Na1	0.5000	0.52321 (14)	0.7500	0.0308 (4)	
P1	0.0000	0.68659 (5)	0.2500	0.00564 (14)	
P2	0.28093 (4)	0.38887 (3)	0.38603 (7)	0.00494 (11)	
O1	0.03662 (11)	0.75858 (10)	0.4624 (2)	0.0078 (2)	
O2	0.10795 (12)	0.61003 (10)	0.2634 (2)	0.0084 (2)	
O3	0.34567 (12)	0.32859 (10)	0.6116 (2)	0.0073 (2)	
O4	0.14051 (11)	0.40754 (10)	0.3420 (2)	0.0085 (2)	
H4	0.1241	0.4816	0.3033	0.013*	
O5	0.28443 (11)	0.32046 (10)	0.1916 (2)	0.0068 (2)	
O6	0.34273 (12)	0.50140 (10)	0.4000 (2)	0.0076 (2)	

### Atomic displacement parameters (Å^2^)

**Table:**

	*U*^11^	*U*^22^	*U*^33^	*U*^12^	*U*^13^	*U*^23^
Mg1	0.0081 (4)	0.0088 (4)	0.0094 (4)	0.000	0.0041 (3)	0.000
Mg2	0.0073 (3)	0.0057 (3)	0.0067 (3)	0.0004 (2)	0.0031 (2)	0.0001 (2)
Na1	0.0118 (6)	0.0691 (11)	0.0091 (6)	0.000	0.0016 (5)	0.000
P1	0.0051 (3)	0.0063 (3)	0.0044 (3)	0.000	0.0007 (2)	0.000
P2	0.0060 (2)	0.00431 (19)	0.0043 (2)	−0.00006 (14)	0.00177 (16)	−0.00011 (14)
O1	0.0063 (6)	0.0110 (5)	0.0054 (5)	−0.0010 (4)	0.0014 (5)	−0.0022 (4)
O2	0.0060 (6)	0.0073 (5)	0.0114 (6)	0.0010 (4)	0.0031 (5)	−0.0007 (4)
O3	0.0086 (6)	0.0080 (5)	0.0049 (5)	0.0020 (4)	0.0024 (5)	0.0016 (4)
O4	0.0073 (6)	0.0058 (5)	0.0131 (6)	0.0009 (4)	0.0048 (5)	0.0004 (5)
O5	0.0077 (6)	0.0077 (5)	0.0052 (5)	0.0003 (4)	0.0028 (5)	−0.0009 (4)
O6	0.0083 (6)	0.0051 (5)	0.0093 (6)	−0.0014 (4)	0.0035 (5)	−0.0002 (4)

### Geometric parameters (Å, º)

**Table:**

Mg1—O3^i^	2.1224 (13)	Na1—O6^x^	2.4386 (13)
Mg1—O3^ii^	2.1224 (13)	Na1—O3	2.8840 (19)
Mg1—O1^iii^	2.1312 (12)	Na1—O3^ix^	2.8840 (19)
Mg1—O1^iv^	2.1312 (12)	Na1—O1^xi^	2.922 (2)
Mg1—O4^v^	2.1669 (14)	Na1—O1^viii^	2.922 (2)
Mg1—O4	2.1669 (14)	P1—O1^v^	1.5372 (12)
Mg2—O6	2.0234 (13)	P1—O1	1.5372 (12)
Mg2—O3^iii^	2.0686 (13)	P1—O2^v^	1.5476 (13)
Mg2—O2	2.0696 (14)	P1—O2	1.5476 (13)
Mg2—O5^vi^	2.0729 (13)	P2—O5	1.5234 (12)
Mg2—O5^vii^	2.0955 (13)	P2—O6	1.5263 (12)
Mg2—O1^viii^	2.1153 (14)	P2—O3	1.5349 (13)
Na1—O6	2.2974 (13)	P2—O4	1.5806 (13)
Na1—O6^ix^	2.2974 (13)	O4—H4	0.9269
Na1—O6^vii^	2.4386 (13)		
			
O3^i^—Mg1—O3^ii^	104.23 (8)	O6^vii^—Na1—O6^x^	166.01 (10)
O3^i^—Mg1—O1^iii^	86.53 (5)	O6—Na1—O3	56.06 (5)
O3^ii^—Mg1—O1^iii^	78.23 (5)	O6^ix^—Na1—O3	111.84 (7)
O3^i^—Mg1—O1^iv^	78.23 (5)	O6^vii^—Na1—O3	62.51 (5)
O3^ii^—Mg1—O1^iv^	86.53 (5)	O6^x^—Na1—O3	105.27 (6)
O1^iii^—Mg1—O1^iv^	155.13 (8)	O6—Na1—O3^ix^	111.84 (7)
O3^i^—Mg1—O4^v^	83.70 (5)	O6^ix^—Na1—O3^ix^	56.06 (5)
O3^ii^—Mg1—O4^v^	170.20 (5)	O6^vii^—Na1—O3^ix^	105.27 (6)
O1^iii^—Mg1—O4^v^	108.38 (5)	O6^x^—Na1—O3^ix^	62.51 (5)
O1^iv^—Mg1—O4^v^	89.53 (5)	O3—Na1—O3^ix^	71.02 (6)
O3^i^—Mg1—O4	170.20 (5)	O6—Na1—O1^xi^	118.48 (6)
O3^ii^—Mg1—O4	83.70 (5)	O6^ix^—Na1—O1^xi^	74.30 (5)
O1^iii^—Mg1—O4	89.53 (5)	O6^vii^—Na1—O1^xi^	84.97 (5)
O1^iv^—Mg1—O4	108.38 (5)	O6^x^—Na1—O1^xi^	107.88 (6)
O4^v^—Mg1—O4	89.05 (7)	O3—Na1—O1^xi^	146.62 (4)
O6—Mg2—O3^iii^	85.88 (5)	O3^ix^—Na1—O1^xi^	129.17 (3)
O6—Mg2—O2	88.80 (5)	O6—Na1—O1^viii^	74.30 (5)
O3^iii^—Mg2—O2	111.03 (6)	O6^ix^—Na1—O1^viii^	118.48 (7)
O6—Mg2—O5^vi^	172.05 (6)	O6^vii^—Na1—O1^viii^	107.88 (6)
O3^iii^—Mg2—O5^vi^	91.58 (5)	O6^x^—Na1—O1^viii^	84.97 (5)
O2—Mg2—O5^vi^	85.07 (5)	O3—Na1—O1^viii^	129.17 (3)
O6—Mg2—O5^vii^	98.45 (5)	O3^ix^—Na1—O1^viii^	146.62 (4)
O3^iii^—Mg2—O5^vii^	162.38 (6)	O1^xi^—Na1—O1^viii^	51.46 (6)
O2—Mg2—O5^vii^	86.23 (5)	O1^v^—P1—O1	111.21 (10)
O5^vi^—Mg2—O5^vii^	86.22 (5)	O1^v^—P1—O2^v^	111.07 (7)
O6—Mg2—O1^viii^	100.87 (6)	O1—P1—O2^v^	108.34 (7)
O3^iii^—Mg2—O1^viii^	79.79 (5)	O1^v^—P1—O2	108.34 (7)
O2—Mg2—O1^viii^	166.18 (6)	O1—P1—O2	111.07 (7)
O5^vi^—Mg2—O1^viii^	86.06 (5)	O2^v^—P1—O2	106.73 (10)
O5^vii^—Mg2—O1^viii^	82.62 (5)	O5—P2—O6	111.03 (7)
O6—Na1—O6^ix^	166.85 (10)	O5—P2—O3	111.42 (7)
O6—Na1—O6^vii^	86.56 (4)	O6—P2—O3	108.82 (7)
O6^ix^—Na1—O6^vii^	91.84 (4)	O5—P2—O4	107.74 (7)
O6—Na1—O6^x^	91.84 (4)	O6—P2—O4	108.99 (7)
O6^ix^—Na1—O6^x^	86.56 (4)	O3—P2—O4	108.78 (7)

Symmetry codes: (i) *x*−1/2, −*y*+1/2, *z*−1/2; (ii) −*x*+1/2, −*y*+1/2, −*z*+1; (iii) *x*, −*y*+1, *z*−1/2; (iv) −*x*, −*y*+1, −*z*+1; (v) −*x*, *y*, −*z*+1/2; (vi) −*x*+1/2, *y*+1/2, −*z*+1/2; (vii) *x*, −*y*+1, *z*+1/2; (viii) −*x*+1/2, −*y*+3/2, −*z*+1; (ix) −*x*+1, *y*, −*z*+3/2; (x) −*x*+1, −*y*+1, −*z*+1; (xi) *x*+1/2, −*y*+3/2, *z*+1/2.

### Hydrogen-bond geometry (Å, º)

**Table:**

*D*—H···*A*	*D*—H	H···*A*	*D*···*A*	*D*—H···*A*
O4—H4···O2	0.93	1.57	2.4932 (17)	174
